# Recall is not necessary for verbal sequence learning

**DOI:** 10.3758/s13421-015-0544-0

**Published:** 2015-08-20

**Authors:** Kristjan Kalm, Dennis Norris

**Affiliations:** MRC Cognition and Brain Sciences Unit, 15 Chaucer Road, Cambridge, CB2 7EF UK

**Keywords:** Recall, Sequence learning, Short-term memory

## Abstract

The question of whether overt recall of to-be-remembered material accelerates learning is important in a wide range of real-world learning settings. In the case of verbal sequence learning, previous research has proposed that recall either is necessary for verbal sequence learning (Cohen & Johansson *Journal of Verbal Learning and Verbal Behavior*, *6*, 139–143, 1967; Cunningham, Healy, & Williams *Journal of Experimental Psychology: Learning, Memory, and Cognition*, *10*, 575–597, 1984), or at least contributes significantly to it (Glass, Krejci, & Goldman *Journal of Memory and Language*, *28*, 189–199, 1989; Oberauer & Meyer *Memory*, *17*, 774–781, 2009). In contrast, here we show that the amount of previous spoken recall does not predict learning and is not necessary for it. We suggest that previous research may have underestimated participants’ learning by using suboptimal performance measures, or by using manual or written recall. However, we show that the amount of spoken recall predicted how much interference from other to-be-remembered sequences would be observed. In fact, spoken recall mediated most of the error learning observed in the task. Our data support the view that the learning of overlapping auditory–verbal sequences is driven by learning the phonological representations and not the articulatory motor responses. However, spoken recall seems to reinforce already learned representations, whether they are correct or incorrect, thus contributing to a participant identifying a specific stimulus as either “learned” or “new” during the presentation phase.

The question of whether overt recall of to-be-remembered material accelerates learning is important in a wide range of real-world learning settings. Here we tackle this question in the context of learning auditory–verbal sequences, a common framework for learning new words, phone numbers, songs, or similar sequences. A standard paradigm for investigating how verbal sequences are learned is the Hebb repetition learning task (Hebb, 1961), in which participants are asked to recall a sequence in the correct order immediately after its presentation. Unbeknownst to participants, every third presented sequence is repeated throughout the experiment. Hebb observed that recall performance for the repeated sequences increased substantially relative to performance of the unique (nonrepeated) sequences. This phenomenon, known as the *Hebb repetition learning effect*, has been replicated many times (see Page & Norris, 2008, for a review). Learning appears to involve the gradual development of a durable representation of both item and order information that facilitates subsequent recognition and recall. Hence, repetition learning of verbal sequences is commonly seen as a model of acquiring new phonological sequences or word learning (Page & Norris, 2008, 2009; Szmalec, Duyck, Vandierendonck, Mata, & Page, 2009). However, one factor that might undermine this claim is that past research has suggested that learning only occurs when the sequences have to be overtly recalled. This finding was first reported by Cohen and Johansson (1967) and later confirmed by Cunningham, Healy, and Williams (1984; see also Glass, Krejci, & Goldman, 1989). In Cohen and Johansson’s experiment participants were told to rehearse the whole of an eight-digit sequence that was grouped into two four-digit chunks, but to recall only one chunk. When participants were not told which chunk was to be recalled until after presentation of the entire sequence, there was no evidence of learning the chunk that had not been repeatedly recalled. Couture, Lafond, and Tremblay (2008) showed that response learning can be solely responsible for the learning effect when sequences are presented auditorily, and that participants learn from their own responses, whether or not the responses are correct.

It is possible that what is learned in these studies is not a phonological representation of the sequence, but rather a motor sequence, analogous to serial reaction time learning (Stadler, 1993). In a paradigm similar to the conventional Hebb task, Glass, Krejci, and Goldman (1989) found that recall measured at the end of the study phase was better when participants had to recall sequences rather than simply to shadow the digits. Interestingly, recognition performance was similar in the two cases. Glass et al. also measured how quickly participants could read visually presented test sequences aloud and found similar improvements in speed for repeated sequences under both presentation conditions. A similar finding was reported by Oberauer and Meyer (2009), who also examined the effects of presentation and recall separately.

There is also evidence that even in a motor task, the representation of the sequence can be learned independently of the motor response itself. Fendrich, Healy, and Bourne (1991) instructed participants to use a keypad to type sequences displayed on a screen. Sequences that were shown several times over a session showed a performance increase over nonrepeating sequences, in terms of speed, accuracy of response, and recognition memory. In a follow-up testing session, the key-to-digit mapping on the keypad was inverted from the standard calculator to a standard telephone layout. The results showed an advantage both for stimuli that had previously been presented but had a new motor response sequence, and for stimuli that were new but shared a previously repeated motor sequence. This double advantage shows that both the sequence and the response were being learned simultaneously. In sum, the respective contributions of phonological and motor learning to verbal sequence learning are unclear.

Here we examined the role of recall in sequence learning under conditions analogous to naturalistic word learning. To this end, we used auditory presentation and spoken recall. Auditory presentation is essential if repetition learning is to be taken as a model of how infants learn new vocabulary. Furthermore, there is no guarantee that findings with visual presentation will generalize to auditory presentation. For example, in addition to the classical findings, such as that auditory presentation produces a more pronounced recency effect than visual presentation (Crowder, 1986; Engle & Mobley, 1976), Conway and Christiansen (2005) found that statistical learning is better with auditory than with visual or tactile stimuli. The latter finding may be particularly important in the present context because the Hebb effect can be seen as a result of learning the statistical properties of the input. If learning is superior with auditory presentation, then even if recall is necessary to produce a Hebb effect with visual presentation, recall might not be necessary with auditory presentation. Spoken recall is essential to ensuring that what is being learned are phonological representations rather than manual sequences. However, the existing data on repetition learning of sequences have been gathered almost exclusively using manual rather than verbal responses (Table 1). Finally, in order to focus specifically on the learning of order rather than item information, our repeating and unique sequences were reorderings of the same set of items.

**Table 1:** Presentation and recall modalities used in previous studies investigating the Hebb effect

Study	Year	Presentation	Response
Hebb	1961	Auditory	Verbal
Melton	1963	Visual, auditory	Manual
Cohen & Johansson	1967	Auditory	Manual
Cohen & Johansson	1967	Auditory	Manual, verbal
Cunningham, Healy, & Williams	1984	Visual	Manual
McKelvie	1987	Auditory	Manual
Fendrich, Healy, & Bourne	1991	Visual	Manual
Cumming, Page, Hitch, & Norris	2003	Visual	Manual
Cumming, Page, Norris, McNeil, & Hitch	2006	Visual	Manual
Conway & Christiansen	2005	Visual	Manual
Page, Cumming, Norris, Hitch, & McNeil	2006	Visual, auditory	Manual
Couture & Tremblay	2006	Visual	Manual
O’Shea & Clegg	2006	Visual	Manual
Couture, Lafond, & Tremblay	2008	Auditory	Manual
Parmentier, Maybery, Huitson, & Jones	2008	Auditory	Manual
Horton, Hay, & Smyth, 2008	2008	Visual	Manual
Oberauer & Meyer	2009	Visual	Manual
Hitch, Flude, & Burgess	2009	Visual, auditory	Manual
Szmalec, Duyck, Vandierendonck, Mata, & Page	2009	Visual	Manual
Lafond, Tremblay, & Parmentier	2010	Auditory	Manual
Szmalec, Page, & Duyck	2012	Visual	Manual
Page, Cumming, Norris, McNeil, & Hitch	2013	Visual	Manual
Kalm, Davis, & Norris	2013	Auditory	Verbal

In this study, we sought to answer the following questions: (1) Is spoken recall necessary for simultaneous learning of overlapping auditory–verbal sequences? (2) Does the amount of previous spoken recall predict (a) the amount of learning on a given sequence and (b) the source of errors for a given sequence, such as intrusions from the competing to-be-learned material (Henson, 1998; Henson, Norris, Page, & Baddeley, 1996). We measured the effect of recall by testing whether the number of previous spoken recalls over a fixed number of repeated presentations predicted the learning of a given auditory–verbal sequence. We also analyzed the sources of errors during learning by matching participants’ responses on a given trial to preceding presentations and responses.

## Method

### Participants

In total, 22 right-handed volunteers (14 female, eight male; 20–33 years old) gave informed written consent for participation in the study after its nature had been explained to them. The participants reported no history of psychiatric or neurological disorders and no current use of any psychoactive medications. The study was approved by the Cambridge Local Research Ethics Committee (Cambridge, UK).

### Procedure

**Fig. 1:**
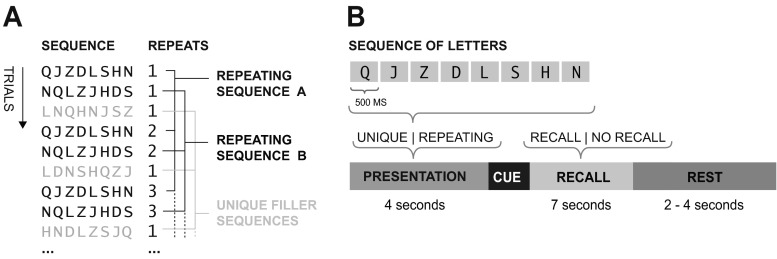
(**a**) Structure of trials. (**b**) A single trial

In our task, participants had to recall sequences of eight auditorily presented monosyllabic letters in the correct order. All sequences consisted of random reorderings of the same eight letters (Q, J, Z, D, L, S, H, and N). The sequences therefore differed only in terms of the order in which the letters were presented. The sequences were constructed subject to the following constraints: There was no positional overlap between consecutive sequences, and all sequences were controlled so as to exclude rhyming letters and meaningful chunks. Sequences either were repeated over the course of the experiment (repeated sequences, repeated 12 times) or were never repeated (unique sequences, presented once). No repeated sequences shared more than two items in the same position. All of the sequences were presented in blocked triplets, in which the first trial was always a unique filler sequence and the last two trials were repeating sequences (Fig. 1).

A new repeating sequence was introduced after the previous sequence had been repeated six times. The first repeating sequence was presented six times during a training session before the experiment to ensure that it had also been presented six times at the start of the experiment. Thus, at any given point in the experiment, two repeating sequences were presented simultaneously, with one of the repeating sequences having been presented fewer than six times, and the other more than six times. This ensured that comparisons between the sequences at different stages of learning were not confounded with time. Existing data had indicated that participants have little difficulty in learning two lists simultaneously, even when those lists are constructed by permuting the same set of items (Kalm, Davis, & Norris, 2013; Saint-Aubin, Guérard, Fiset, & Losier, 2015).

To distinguish between repetition learning with and without recall, we intermixed the trials with and without overt recall, so that response learning either fell to the beginning or the end of the repetitions of a given sequence. For half of the repeating sequences participants recalled eight trials out of 12 (Repetitions 1–4 and 7–10; “recall-early” sequences) and, for the other half, they recalled four trials (Repetitions 4–5 and 10–11; “recall-late” sequences; see Table 2). Therefore, the fourth and tenth presentations of both types of repeated sequences allowed us to compare how performance depends on how often the sequence was previously recalled. This procedure also meant that the amount of data from sequences’ repetitions varied: The recall scores for Repetitions 1, 2, 3, 7, 8, and 9 came exclusively from recall-early sequences, and the recall scores for Repetitions 5 and 11 came from recall-late sequences. There were no scores for Repetitions 6 and 12. To counterbalance the resulting inequalities in overall recall condition, and to keep the trial structure unpredictable for the participants, we also manipulated the recall of intermediate unique sequences.

**Table 2:** Experiment structure: Numbers of recalled trials for repeating sequences according to repetition number (E = recall-early sequence, L = recall-late sequence)

Repetition	1	2	3	4	5	6	7	8	9	10	11	12
Recalls	12	12	12	24	12	0	12	12	12	24	12	0
Sequence type	E	E	E	E + L	L	–	E	E	E	E + L	L	–

On each trial, participants were presented with a visual fixation cross to indicate the start of the auditory presentation of the sequence. Eight letters were then presented at a rate of 500 ms per item, followed by a “?” cue, indicating that they were to verbally recall the sequence exactly as they had just heard it, or a “--” cue, indicating that they should not respond. They then had to wait 2–4 s for the next sequence (Fig. 1). The letters were spoken by a native English-speaking male and recorded at a 44.1-kHz sampling rate and 16 bits per sample. Recordings were made in a soundproof room, and the perceptual center of the syllable was synchronized to a common onset time such that sequences were heard to be rhythmic. This enabled us to control for the time difference in pronouncing different letters. In sum, each participant was presented with 216 trials in addition to an initial practice session. The participants were not informed that there were different types of trials.

### Recall performance evaluation

#### Learning effects

The standard method for scoring serial recall is only to consider an item correct if it is recalled in the same serial position where it appeared in the input. However, with spoken recall in particular, participants often omit items. If a participant omits the first item in a sequence and recalls the remaining items in the correct order, their response would be scored as completely incorrect. The participant thus receives no credit for recalling most of the items in the correct order. A similar problem arises when measuring learning. If participants learn a subsequence of the items accurately, but fail to recall the start of that sequence in the correct position, the score would not capture the fact that some learning has clearly taken place. To overcome this problem, our primary measure of recall was based on a Levenshtein (1966) edit distance, which corresponds to the smallest number of edit operations that are necessary to modify one string in order to obtain another string, where an operation is defined as the insertion, deletion, or substitution of a single character. Any two identical strings will have a Levenshtein distance of 0. If one item is omitted from anywhere in a sequence, the distance will be 1. If a sequence is recalled in reverse order, the Levenshtein distance will be the same as the length of the sequence. Although we had clear pragmatic reasons for using an edit-distance scoring procedure, there are theoretical reasons too. Strict in-position scoring carries with it an implicit assumption that what is being learned is something like position–item associations. On the other hand, if what is being learned is order representations, such as chunks or item–item associations, then one would expect that partially learned sequences might contain subsequences that would be displaced from their original positions. In line with this, Chen and Cowan (2009) noted that strict positional scoring may underestimate the benefit of chunking. The Levenshtein scoring procedure is theory-neutral, since it gives credit for partial learning. For comparison with the previous literature we analyzed our data using both an edit-distance metric and the more conventional correct-in-position strict serial-recall procedure. An illustration of how the Levenshtein edit distance is computed is given in the Appendix. Other examples can be found in Norris and Kinoshita (2012).

Here we used a derived Levenshtein edit-distance metric introduced by Kalm, Davis, and Norris (2013) to produce a score analogous to proportion correct. Two identical sequences have an edit distance of 0, whereas two completely different sequences have a score given by the length of the sequence. To compute our derived score we divided the edit distance by the length of the sequence and subtracted this from 1. Thus, the recall score was computed as follows:

$$ \mathrm{Score}=1-\kern0.5em \left[ Levenshtein\_ distance\kern0.5em \left(``\mathrm{presented}\ \mathrm{string},"\ ``\mathrm{recalled}\ \mathrm{string}"\right)\kern0.5em /\kern0.5em \left(\mathrm{number}\ \mathrm{o}\mathrm{f}\ \mathrm{letters}\ \mathrm{t}\mathrm{o}\ \mathrm{recall}\right)\right]. $$

The resulting Levenshtein distance (henceforth, LD) score takes the maximum value of 1 if all items were recalled in their original serial positions. Note that any two randomly generated strings would tend to have a Levenshtein distance that was less than the length of the sequence. In the case of eight-item sequences, the expected Levenshtein distance between randomly generated strings would be 6.3, and the LD score would be .21.

#### The effect of recall

To capture the effect of previous recalls and the sources of errors in recall, we calculated two additional Levenshtein distances. First, we calculated a separate Levenshtein distance to match recall with previous presentations (henceforth, LDP). This was done by matching the response on trial *n* to all of the previous *m* presentations preceding trial *n*, including both repeating and unique sequences. As a result, the LDP for trial *n* is the greatest LD between the current response and the *m* previous presentations:

$$ \mathrm{L}\mathrm{D}\mathrm{P}\kern0.75em =\kern0.5em  arg\  max\left[\mathrm{L}\mathrm{D}\left({\mathrm{R}}_n,\ {{\mathrm{P}}_n}_{\hbox{--} 1}\right),\ .\ .\ .\ \mathrm{L}\mathrm{D}\left({\mathrm{R}}_n,\ {{\mathrm{P}}_n}_{\hbox{--} m}\right)\right], $$

where LD(R, P) is the Levenshtein distance between recall and presentation, and *m* is a history parameter determining how many previous presentations are taken into account. (Note that the Levenshtein distance between two recalled strings is divided by the number of letters presented on the trial, not the number of letters in the recalled string.)

Similarly, an additional Levenshtein distance, sensitive to past recalls (LDR), was calculated by matching the response on trial *n* to all of the previous *m* recalls preceding trial *n*, including both repeating and unique sequences:

$$ \mathrm{L}\mathrm{D}\mathrm{R}\kern0.75em =\kern0.5em  arg\  max\left[\mathrm{L}\mathrm{D}\left({\mathrm{R}}_n,\ {{\mathrm{R}}_n}_{\hbox{--} 1}\right),\ .\ .\ .\ \mathrm{L}\mathrm{D}\left({\mathrm{R}}_n,\ {{\mathrm{R}}_n}_{\hbox{--} m}\right)\right]. $$

It follows that for any trial *n*, the LDP and LDR scores can only be higher than the LD score if the recall on that trial is a better match with some previous presentation or recall. Thus, we interpreted the differences between the standard and modified Levenshtein distances on trial *n* as the amounts of interference from past presentations and past recalls, respectively. Thus, we calculated interference using the following equations: interference on trial *n* from past presentations, IP_*n*_ = LDP_*n*_ − LD_*n*_; interference on trial *n* from past recalls, IR_*n*_ = LDR_*n*_ − LD_*n*_.

## Results

**Fig. 2:**
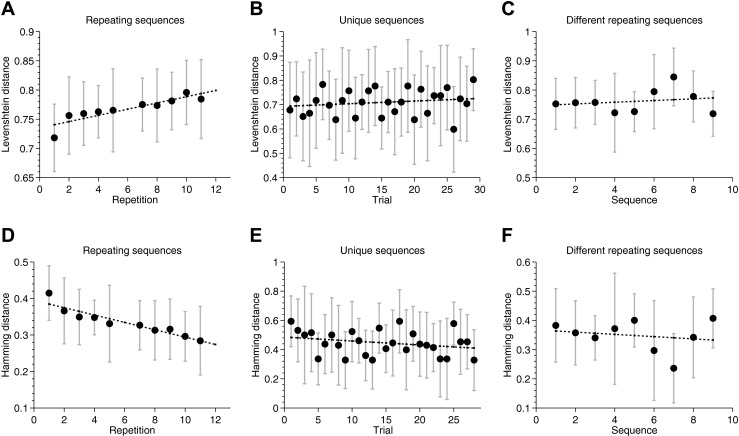
Performance on repeating, filler, and different repeating sequences, measured with Levenshtein distance (**a**–**c**, respectively) and positional scoring (**d**–**f**, respectively). Behavioral performance data are shown only for trials on which recall was measured, and error bars show *SEM*s for the variability across participants. Note that the Levenshtein and positional scores are not directly comparable and scalable, despite both being normalized between 0 and 1. This is because the Levenshtein distance is a similarity metric and, given common items between two strings, can never be zero, unlike the positional score

To establish the main effect of learning over repetitions, performance must be shown to increase for a repeated sequence relative to nonrepeated controls (unique filler sequences). Hence, the slopes of immediate serial recall performance over the course of the experiment were calculated using least-squares linear regression for the repeating and unique sequences for every participant. A paired *t*-test over the participants’ slopes for repeated and filler sequences showed a significant Hebb effect [*t*(21) = 3.29, *p* < .003]. Separate one-sample *t*-tests showed that the slope of repeating sequences was significantly different from zero [*t*(21) = 5.81, *p* < .001; Fig. 2a], whereas the slope for filler sequences was not [*t*(21) = 0.52, *p* = .31; Fig. 2b]. We observed no significant change in recall performance across different repeating sequences (Fig. 2c).

When recall performance was measured with strict positional scoring, we found no sign of learning. In fact, a paired *t*-test over the participants’ slopes showed a consistent *decrease* in recall performance across repetitions [*t*(21) = –3.29, *p* < .003; Fig. 2d], and the slopes of filler sequences were not significantly different from zero [*t*(21) = 0.52, *p* = .31; Fig. 2e]. The results show that a significant Hebb effect was only revealed with similarity-based scoring, but not with strict positional scoring.

Next we sought to determine whether spoken recall was necessary for verbal sequence learning and whether the number of previous spoken recalls predicted the amount of learning. For this purpose we measured memory performance on both the recall-early and recall-late types of sequences after four and ten repeated presentations. At the fourth presentation recall-early sequences had previously been recalled three times, whereas recall-late sequences had not been recalled at all. At the tenth presentation, recall-early sequences had been previously recalled seven times and recall-late sequences two times.

**Fig. 3:**
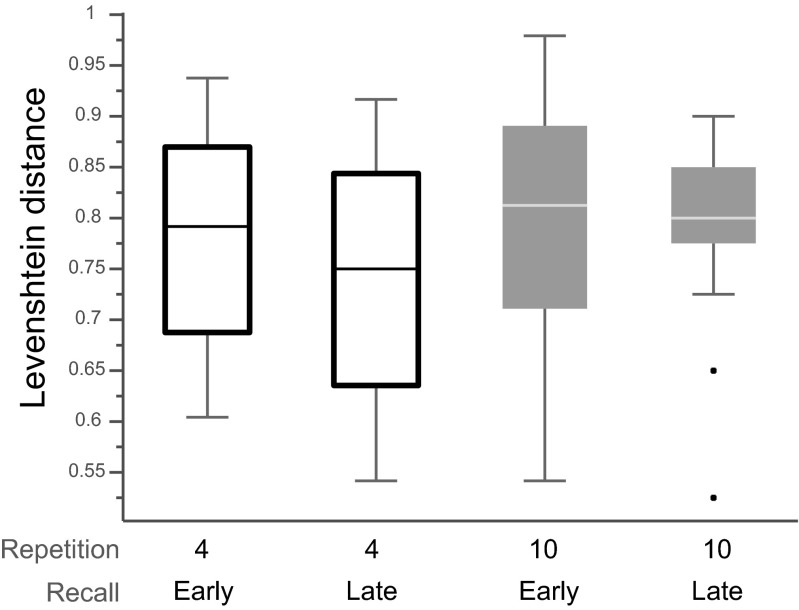
Effects of previous recall. In each box, the central mark is the median, the edges of the box are the 25th and 75th percentiles, and the whiskers extend to the most extreme data points not considered outliers

We observed no effect of previous recall at either the fourth or the tenth repetition [fourth position, *t*(21) = 0.87, *p* = .39; tenth position, *t*(21) = 0.81, *p* = .43; Fig. 3]. However, at both the fourth and tenth repetitions of both types of repeating sequences participants’ memory performance was significantly better than their performance on filler sequences [fourth position, *t*(21) = 4.21, *p* < .002; tenth position, *t*(21) = 5.01, *p* = .001]. The results show that participants’ memory performance was not significantly better for previously recalled sequences than for sequences that had not or had little been recalled before. Hence, in our task previous spoken recall is not necessary to learn overlapping auditory–verbal sequences, and the amount of previous spoken recall does not predict the amount of learning on a given sequence.

Next, we sought to establish whether previous spoken recall predicted the source of errors for a given sequence, such as intrusions from the competing to-be-learned material (Henson et al., 1996). For every participant’s response, we calculated measures of interference from the eight previous presentations and from the participant’s own responses. These measures of interference were based on the assumption that if an erroneous recall of sequence *S* had a better Levenshtein match with another previously presented sequence, *M*, we could hypothesize that the error was caused by the interference from sequence *M* on sequence *S* (for a full description of calculating the interference measures, see the Recall Performance Evaluation section above).

**Fig. 4:**
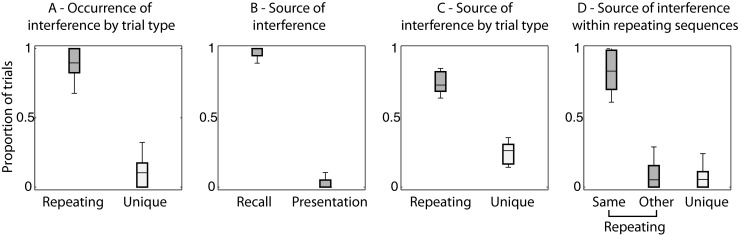
Analysis of interference. In each box, the central mark is the median, the edges of the box are the 25th and 75th percentiles, and the whiskers extend to the most extreme data points not considered outliers

The average proportion of trials on which interference was detected was approximately 10 % across participants. The vast majority of the interference was observed on trials with repeating sequences (mean 89.5 %) [*t*(42) = 24.15, *p* < .001; Fig. 4a]. To identify the source of interference, we looked at whether the interfering sequence was a previous presentation or recall (the participant’s own response), and the type of interfering sequence (repeating or unique). The vast majority of interference came from previous recalls (mean 97 %) [*t*(42) = 64.2, *p* < .001; Fig. 4b], not presentations (3 %). In terms of sequence types, the source of interference was mostly other repeating sequences (75 %) [*t*(42) = 20.9, *p* < .001; Fig. 4c] rather than unique ones (25 %). When looking at the source of interference within repeating sequences we observed that most of the interference came from previous trials of the same repeating sequence itself [same vs. other repeating sequences, *t*(42) = 20.32, *p* < .001; same repeating sequence vs. unique sequences, *t*(42) = 21.38, *p* < .001; Fig. 4d].

**Fig. 5:**
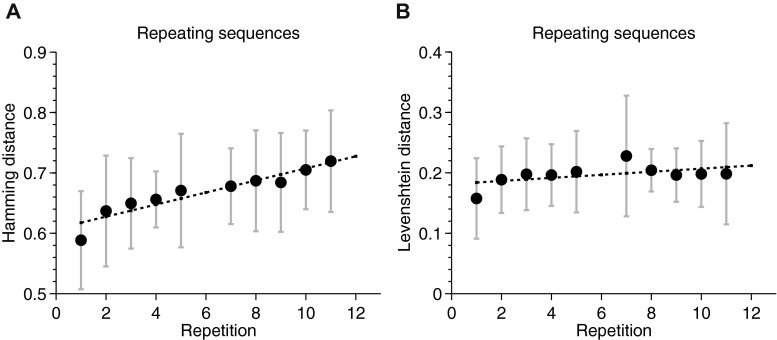
Proportions of trials with interference from previous recalls and presentations. Interference measured with (**a**) positional scoring and (**b**) Levenshtein distance. Performance data are only shown for trials on which recall was measured. Error bars show *SEM*s for the variability across participants

When strict positional scoring was used, the proportion of trials on which interference was detected was approximately 60 % across participants (as compared to approximately 10 % when Levenshtein scoring was used). Furthermore, plotting the amount of interference against repetitions showed a gradual and significant increase in interference across the experiment (Fig. 5a), which was not significant when interference was measured with the Levenshtein distance (Fig. 5b).

We also analyzed the pattern of interference from previous trials using the procedure described by Couture et al. (2008). This produced qualitatively similar results; that is, both scoring methods revealed evidence of response learning. However, we do not report the details of this analysis, since is not well suited to the treatment of spoken serial recall because it assumes that participants do not make omissions. Couture et al. used manual recall, in which participants were not permitted to make omissions.

In summary, with edit-distance scoring we observed learning independently of whether or not participants recalled the repeating sequences overtly. However, at the same time, recall did seem to be a source of interference. Interference most frequently arose from responses given to preceding trials rather than from the sequences actually presented. Furthermore, most of the interference came from responses given to repeated sequences. This contrasted with the pattern observed with strict positional scoring, in which we found no sign of any improvement in performance over repetitions and in which six times as many trials showed signs of interference as with edit-distance scoring.

Although interference was measured by comparing the response on the current trial with the response on previous trials, we should be cautious in attributing this interference to the responses themselves; participants’ responses are simply the only measure we have of what participants have learned. On no-recall trials, participants’ memory may have been exactly the same. It might be more appropriate to take this as an effect of interference between memories for different sequences.

The finding that recall is not necessary in order to produce a Hebb effect is consistent with the two main computational models of the Hebb task—Page and Norris (2009) and Burgess and Hitch (2006). As currently formulated, both models learn only from presentation and not from recall. Saint-Aubin, Guérard, Fiset, and Losier (2015) described how both models are also capable of learning more than one list at a time. Indeed, Page and Norris (2009) presented simulations of learning two lists. One concern might be whether the models might suffer from proactive interference as successive new lists are introduced. This would certainly be the case, but in both models the interference would be short-lived. Note that in the Page and Norris (2009) model, once learning has progressed to a sufficient level, sequence nodes can become “committed” to that specific sequence and will be protected from interference.

## Discussion

Our interest in sequence learning with auditory presentation stems from the view that the Hebb effect can be considered to be a laboratory analogue of phonological word-form learning (Page & Norris, 2008, 2009). If learning in the Hebb paradigm required overt repetition, this would undermine its value, because infants acquire phonological and vocabulary knowledge prior to being able to accurately repeat what is spoken to them. However, previous research on the Hebb task had reported that repeating sequences are not learned in the absence of recall (Cohen & Johansson, 1967; Cunningham et al., 1984). Furthermore, the benefits of recall appear to be partially offset by the fact that participants tend to repeat previous erroneous responses (Couture et al., 2008). However, previous studies have almost exclusively used visually presented lists combined with manual recall, and therefore the data may not have any direct bearing on the value of the Hebb task as a model of word learning. A second potential problem with previous studies is that they all used a strict serial-recall scoring; that is, the participant’s memory for a given sequence was measured by how many items were recalled at their original position in the sequence (Cohen & Johansson, 1967; Cunningham et al., 1984; Fendrich et al., 1991; Melton, 1963; Schwartz & Bryden, 1971). No credit was given for any component of the sequence that was not recalled in exactly the correct serial position. Indeed, this is the scoring procedure used for the vast majority of studies of verbal short-term memory. Such a scoring method has been shown to significantly underestimate the true effects of learning (Couture et al., 2008; Kalm et al., 2013). For example, Cohen and Johansson showed that despite the lack of learning in their no-recall condition, participants still reliably recognized the repeating sequences as familiar; that is, they had learned something about the sequences. This suggests that participants might possibly have learned partial information about the sequence that was not picked up by strict position scoring. Partial learning can potentially be detected by measures of string similarity. Here we used a Levenshtein edit-distance metric. The Levenshtein metric makes only the most minimal assumption about what is being learned. It simply assumes that, as learning progresses, recall should become more and more similar to the presented sequence. As we noted in the introduction, there was a simple pragmatic reason for using an edit-distance metric: With verbal recall participants frequently omit responses. Using positional scoring a single omission at the start of a sequence could move all subsequent items out of position, resulting in a score of 0, even though participants clearly have some knowledge of the relative ordering of the items recalled. However, we had a far more important theoretical reason for using the edit-distance scoring procedure: Position scoring carries the implicit assumption that what is being learned is something akin to position–item associations. If, instead, participants gradually accumulate knowledge about the relative order of items in the sequence or about chunks in the sequence, there would be no reason to expect that those items would necessarily be recalled in the correct absolute position, but such learning would be captured by the Levenshtein score.

The difference that we observed between edit-distance scoring and strict positional scoring raises two important questions: What is being learned, and what counts as an error? Couture, Lafond, and Tremblay (2008) presented a detailed analysis of the buildup of errors in a Hebb paradigm using positional scoring. They focused on errors in which an item was displaced by one position, and showed that such errors tended to be repeated in subsequent sequences. They interpreted these repeated errors as positional protrusions and took this as evidence that participants tended to learn their erroneous responses. However, consider the case in which participants might successfully learn a subsequence of several items, but that sequence is displaced by one position. Their positional recall score would then be 0. If that subsequence is recalled again on later trials, all items in that subsequence would be scored as positional protrusions. However, these protrusions are only positional under the assumption that what is being learned is position–item associations. Our edit-distance analysis of error learning also revealed that participants tended to learn erroneous responses; however, there was no implication that those errors were positional.

In the same way that positional scoring may underestimate how much has been learned and give a misleading impression of what has been learned, it may also underestimate the rate of learning. If one extra item in a subsequence were recalled after every presentation, this would be taken as evidence that participants were only learning from their errors. But this interpretation again depends on the assumption that sequence knowledge is acquired by learning position–item associations. In other words, with position scoring, learning about the relative order of items counts for nothing unless those items are recalled in the correct position. In contrast, according to any other view, this would be taken as evidence that participants were making progress toward learning the sequence correctly. This improvement is captured by using an edit-distance metric. The Levenshtein recall score will improve as more items in the sequence are learned. Moreover, this improvement would no longer be taken as evidence of error learning. According to the Levenshtein distance measure, responses are only attributed to error learning when recall is more similar to recall of some other sequence than it is to the current sequence. In the light of these differences between the scoring procedures, it is not too surprising that with positional scoring our participants showed no signs of learning, only interference. However, edit-distance scoring revealed that they were gradually accumulating useful and accurate knowledge about the sequence of items. The discrepancy between positional and edit-distance scoring shows that what is being learned is more than just position–item associations; participants also accumulate partial knowledge of subsequences or chunks of the sequence, and it makes no sense to classify this partial knowledge as errors. Importantly, this learning takes place even in the absence of overt recall.

## Figures and Tables

**Fig. A1:**
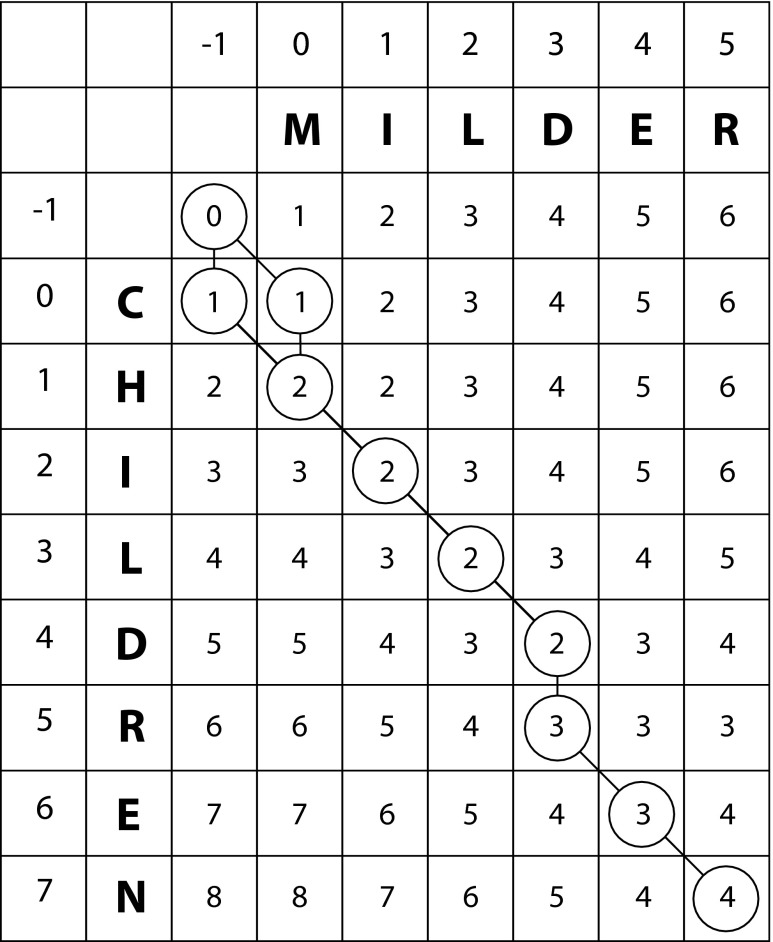
Calculating Levenshtein distance with a dynamic programming approach. An (*m*, *n*) matrix is initialized to measure the Levenshtein distance between the *m*-character string “children” and the *n*-character string “milder.” The matrix can be filled from the upper left to the lower right corner. Each jump horizontally or vertically corresponds to an insertion or a deletion and increases the distance by 1. A diagonal jump increases the distance by 1, if the two characters in the row and column do not match, or by 0, if they do. The number in the lower right corner is the Levenshtein distance between the two strings, since each cell of the matrix minimizes the distance locally

## Appendix: Example of Levenshtein distance calculation

The Levenshtein distance between two strings is defined as the minimum number of edits needed to transform one string into the other, with the allowable edit operations being the insertion, deletion, or substitution of a single character. The Levenshtein edit distance between two strings “minor” and “major” is 2, since the following two edits change one into the other, and there is no way to do it with fewer than two edits:

1. major → mijor (substitution of “a” with “i”)
2. mijor → minor (substitution of “j” with “n”)

In less trivial cases, the Levenshtein distance is sensitive to omissions. Converting a string “children” to “milder” takes at least four edits:

1. “children” → “mhildren” (substitution: “c,” “m”)
2. “mhildren” → “m_ildren” (deletion/omission: “h”)
3. “mildren” → “mild_en” (deletion/omission: “r”)
4. “milden” → “milder” (substitution: “n,” “r”)

Thus, the algorithm produces a matrix (Fig. A1) in which the resulting Levenshtein score is the sum of the minimum-edit costs (the bottom-right element). Note that several alternative paths may all produce the same score.
